# Factors associated with perceived social support among adolescents in Gamo Zone, Southern Ethiopia: a community-based cross-sectional study

**DOI:** 10.3389/fpsyt.2024.1429886

**Published:** 2024-10-18

**Authors:** Negussie Boti Sidamo, Amene Abebe Kerbo, Yohannes Dibaba Wado, Mengistu Meskele Koyira, Kassa Daka Gidebo

**Affiliations:** ^1^ School of Public Health, College of Medicine and Health Sciences, Arba Minch University, Arba Minch, Ethiopia; ^2^ School of Public Health, College of Health Sciences and Medicine, Wolaita Sodo University, Wolaita Sodo, Ethiopia; ^3^ African Populations and Health Research Center, Nairobi, Kenya

**Keywords:** perceived social support, adolescents, cross-sectional study, community-based, mental well-being, Gamo Zone, Southern Ethiopia

## Abstract

**Introduction:**

Perceived social support plays a crucial role in adolescent development, health, well-being, and resilience. Understanding the factors associated with perceived social support among adolescents is essential for designing effective interventions. However, research in this area, particularly within specific contexts, remains limited. Therefore, this study aims to identify the factors associated with perceived social support among adolescents in the Gamo Zone, South Ethiopia Regional State.

**Method:**

A community-based cross-sectional study was conducted, involving 1172 adolescents selected through a stratified multi-stage sampling method. Structured face-to-face interviews were employed for data collection. Summary statistics were utilized for data presentation. Multiple linear regressions were then performed to identify factors associated with perceived social support. The strength and direction of associations were presented using β coefficients and 95% confidence intervals (CIs). The level of statistical significance was set at alpha 5%.

**Result:**

The total mean for perceived social support among adolescents was 57.02 ± 12.68. Adolescents living with their biological parents reported significantly higher levels of perceived social support (β = 4.17, 95% CI: 2.00 to 6.34) compared to their counterparts. Similarly, adolescents engaged in paid work within the last 12 months reported higher perceived social support (β = 3.43, 95% CI: 1.39 to 5.46). Higher levels of parental monitoring were also associated with increased perceived social support (β = 3.03, 95% CI: 1.23 to 4.82). Additionally, adolescents who perceived risks for sexual and reproductive health (SRH) problems reported higher levels of perceived social support (β = 2.76, 95% CI: 0.84 to 4.69). Moreover, adolescents with good knowledge about SRH rights exhibited increased perceived social support (β = 2.46, 95% CI: 0.89 to 4.02). Furthermore, adolescents residing in rural areas reported higher levels of perceived social support compared to those in urban areas (β = 1.56, 95% CI: 0.16 to 3.11).

**Conclusion:**

The findings of this study reveal that factors such as living arrangements, employment status, parental monitoring, perceived risks for SRH problems, knowledge about SRH rights, and geographical context emerged as important predictors of perceived social support. Therefore, implementing interventions and initiatives to address these identified factors holds promise for nurturing resilient adolescent social support networks.

## Introduction

Adolescence is a critical developmental period characterized by significant physical, cognitive, emotional, and social changes (1). This transitional period, typically covering from ages 10 to 19 years of age, is marked by advanced patterns of thinking and reasoning, heightened exploration, identity formation, and the establishment of autonomy, forming new social relationships and attachments, and developing an increasing sense of responsibility and independence (2).

Social support plays a central role in the transition from childhood to adulthood and is widely recognized as a fundamental determinant of adolescent well-being (3). Perceived social support is defined as the subjective perception of being cared for, valued, and supported by others. It encompasses a spectrum of support, encouragement, and companionship perceived as accessible from one’s social circles (4). Adolescents actively seek out connections with peers, family members, and other significant individuals as they strive to understand themselves and their place in the world (5). Numerous studies have highlighted the importance of perceived social support in protecting against the negative impact of stressors and promoting positive adjustment during adolescence (6–8). Adolescents who perceive higher levels of social support tend to show better mental health outcomes, including lower levels of depression, anxiety, and loneliness, as well as higher self-esteem and overall life satisfaction (9).

Despite the growing recognition of the crucial role perceived social support plays in adolescent development (10–12), there remains a notable research gap concerning the specific factors influencing perceived social support among adolescents. Many prior studies have predominantly focused on adults living with HIV/AIDS who attend ART clinics (13–15). The lack of clarity regarding the determinants of perceived social support among adolescents hinders the ability to effectively address adolescents’ social and emotional needs. Without a comprehensive understanding of these factors, interventions and support systems may fail to adequately target the underlying mechanisms that influence adolescents’ perceptions of support. Now more than ever, focused research on the perceived social support of adolescents is urgently needed to fill gaps in data and inform successful programs and policies to meet the needs and fulfill the rights of adolescents (7, 16).

Therefore, this study aimed to identify factors associated with perceived social support among adolescents, to provide insights that contribute to a deeper understanding of adolescent development and well-being. By comprehensively examining the determinant factors of perceived social support, this study aims to generate evidence-based recommendations for fostering supportive environments for adolescent development. Understanding the factors influencing adolescents’ perceptions of social support is paramount. Firstly, it offers valuable insights for designing targeted interventions and support systems that effectively address adolescents’ social and emotional needs. Furthermore, this study holds significance in informing the development of evidence-based interventions and policies aimed at promoting adolescent well-being. By identifying modifiable factors that influence perceived social support, policymakers and practitioners can implement strategies to strengthen adolescents’ support networks and enhance their resilience in the face of adversity. Ultimately, this study aims to enrich the ongoing dialogue surrounding adolescent development and social support, providing actionable insights for research, practical applications, and policy formulation. By elucidating the factors associated with perceived social support among adolescents, this research strives to foster supportive environments that nurture the holistic well-being of adolescents, thereby laying the foundation for healthy and thriving communities.

## Theoretical framework overview

Our study on factors associated with perceived social support among adolescents in the Gamo Zone, South Ethiopia, is guided by Social Cognitive Theory (SCT). SCT, originally developed by Bandura (1986), emphasizes the dynamic interplay between personal factors, environmental influences, and behaviors in shaping social outcomes (17). This framework guided our measurement of perceived social support by focusing on how adolescents’ interactions within their social networks and their perceptions of these relationships impact their overall support systems.

SCT has been widely applied in adolescent health research, particularly in understanding social behavior and support networks. For example, a study by Luszczynska and Schwarzer, 2015 (18), demonstrated that SCT effectively explains the influence of social and environmental factors on adolescents’ health behaviors, including perceived social support. Additionally, empirical evidence from sub-Saharan Africa, such as the research conducted by Njau et al., 2021 (19), supports the applicability of SCT in examining how adolescents’ social environments contribute to their perceived support. These studies emphasize that SCT is particularly relevant in resource-constrained settings like Ethiopia, where social structures and community interactions play a crucial role in shaping health outcomes.

By employing SCT, our study used validated measurement tools that align with the theory’s emphasis on the interaction between individual and environmental factors. This approach ensures a comprehensive understanding of perceived social support among adolescents in the Gamo Zone, aligning with the established theoretical and empirical frameworks that underscore the significance of social cognitive processes in adolescent health.

## Materials and methods

### Study setting and design

**Figure 1:**
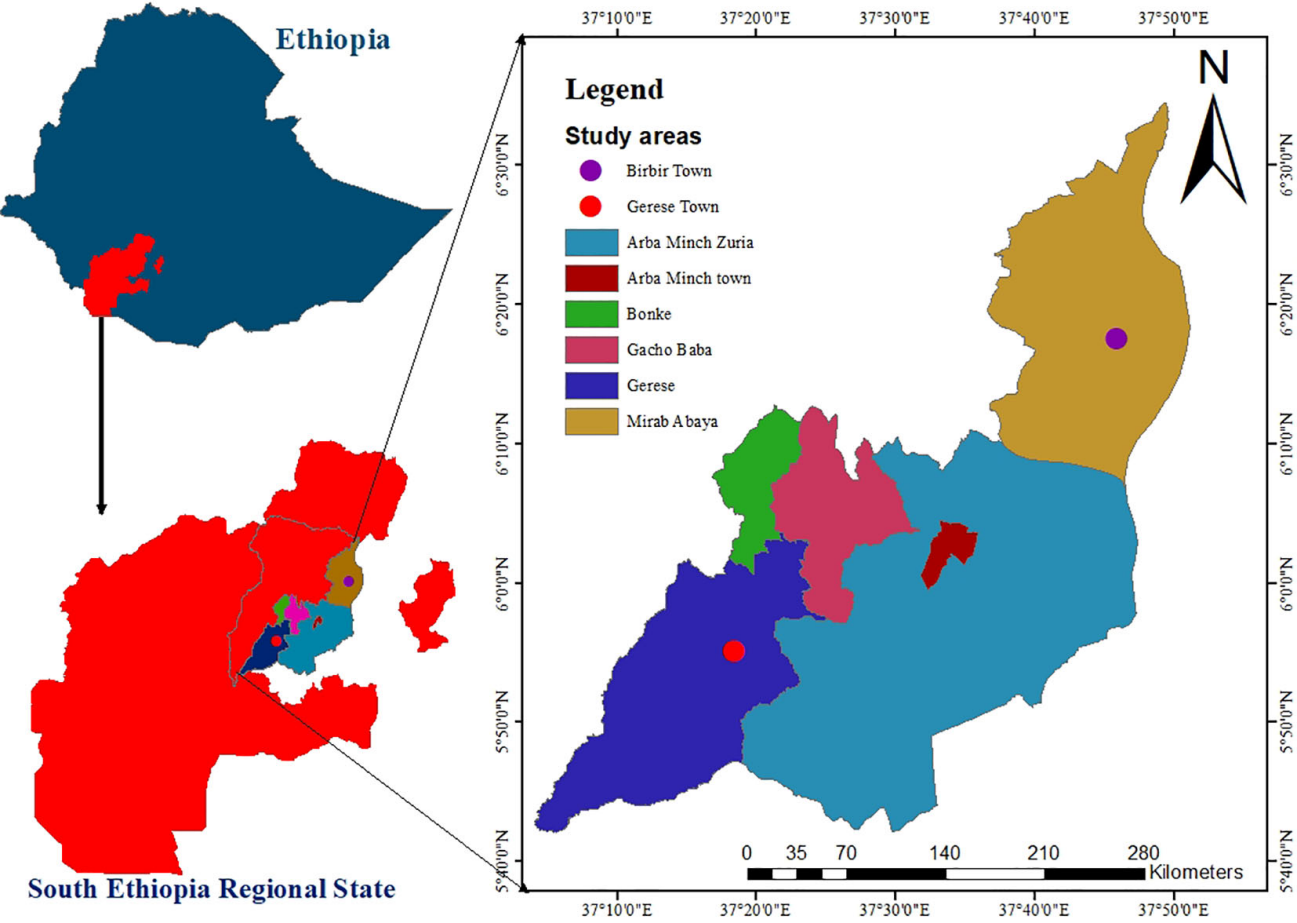
Map of the study area.

A community-based cross-sectional study was undertaken from March 2 to April 9, 2023, in the *Gamo Zone.* This *zone* is one of the zones in the Southern Regional State of Ethiopia. Administratively Ethiopia is divided into 4 levels: the first level (*regions*), the second (*zones*), the third *(woredas)*, and *kebeles* (the lowest administrative level) (20). Arba Minch town is the administrative center of this Zone. This town is located 431 km from the Ethiopian capital city (Addis Ababa). Six town administrations and 14 rural districts with 306 *kebeles* were found in the Gamo zone (**Figure 1**).

This map depicts the study area, highlighting key geographic features, administrative boundaries, and the distinction between urban and rural regions. The areas marked correspond to the locations where data collection was conducted.

### Population

All adolescents in the Gamo zone were the source population. All randomly selected adolescents in the selected study area during the study period and fulfilled the inclusion criteria were the study population.

### Eligibility criteria

The inclusion criteria are adolescents aged 10–19 years and permanent residents (living for more than 6 months) in the selected *kebeles*, and living with their parents or caregivers were eligible for this study. However, those adolescents who had a known hearing or mental impairment and/or were seriously ill at the time of data collection were excluded from this study.

### Sample size determination

The sample size for this study was determined using G*Power software, incorporating several key assumptions to ensure statistical robustness. We selected an effect size of 0.15, which is classified as a medium effect size according to Cohen’s criteria (21). This indicates that we expect the independent variables to explain approximately 15% of the variance in the dependent variable. Medium effect sizes are commonly utilized in social science research as they offer a balanced perspective between overly optimistic and conservative estimates, thereby providing both practical and meaningful insights.

The alpha level was set at 0.05, representing a 5% risk of Type I error. This significance threshold is standard in statistical testing and is widely used to ensure that findings are statistically significant while controlling for false positives.

We opted for a power level of 0.80, which signifies an 80% probability of detecting a true effect if one exists. This level of power is a conventional standard in research, effectively balancing the risk of Type II errors (failing to detect a true effect) with practical sample size considerations (21). An 80% power ensures that the study is sufficiently sensitive to detect significant effects without necessitating an excessively large sample size.

Given the complexity of our regression model, which includes nine predictors, we adjusted the sample size to accommodate this number. The inclusion of multiple predictors generally requires a larger sample size to maintain statistical power (22).

Based on these parameters, G*Power calculated that a sample size of 114 participants is necessary. This sample size aligns with the desired power and significance level while considering the medium effect size and the number of predictors. Collecting data from 114 participants will provide a robust foundation for our regression analysis, ensuring both statistical rigor and practical feasibility.

However, as this study is part of a broader Ph.D. dissertation focused on adolescent health in Southern Ethiopia, a larger sample size was calculated for another objective within the dissertation related to Adolescent Sexual and Reproductive Health (ASRH) service utilization (23). This objective required a sample size of 1,181 participants. Since both studies target the same population and geographic area, we have opted to use the larger sample size of 1,181 participants to maintain consistency and coherence in the sampling framework.

The ASRH study employed rigorous sample size calculations to ensure adequate statistical power for detecting significant associations related to adolescent health outcomes. By applying the same sample size to the current study on adolescent social support, we ensure methodological consistency and comparability of findings across both studies. This integrated approach enhances the overall understanding of adolescent health issues in the region, promotes resource efficiency, and upholds the scientific rigor required for a comprehensive doctoral dissertation.

### Sampling technique

A multistage stratified sampling technique was used to select a representative sample of adolescents. In the first stage, the Gamo zone was stratified into urban and rural administrative strata. To ensure the precision of the survey in each stratum (urban vs. rural), an equal number of samples (primary sampling unit) were selected. This approach is commonly used in demographic surveys in Ethiopia (27). The use of implicit stratification and proportional allocation was achieved at each lower administrative level *(Kebele)* by sorting the sampling frame within each sampling stratum before sample selection (27). Three of the fourteen rural districts and three of the six town administrations were selected by lottery. In the second stage, 36 *kebeles*(11 urban *kebeles* and 25 rural *kebeles*) were selected with probability proportional to the *kebeles* in each stratum and with independent selection from each sampling stratum. Households with eligible participants (adolescents) were the third-stage sampling units sampled from the selected *kebeles*. Then the sample size was proportionally distributed among each of the selected *kebeles.* With the assistance of a health extension worker, sampling frames were created for each selected *kebele* using the family logbook. Then households who had eligible adolescents were selected using a simple random sampling technique. Finally, adolescents (10–19 years old) who were present in the household at the time of the visit and agreed to participate were interviewed. If there is, more than one eligible adolescent in the selected household, one was selected by lottery method.

### Measurement

#### Perceived social support

The questions are adapted from the revised version of the Perceived Social Support measure of the multi-dimensional scale (the revised MSPSS). The revised MSPSS scale had 12-item questions to assess the adolescents’ perceived support from friends, family, and significant others. Every item uses a seven-point Likert scale ranging from 1 (very strongly disagree) to 7 (very strongly agree) (28, 29). There are three subscales used by the scale: Significant Others (SO) (Items 1, 2, 5, and 10), Family (FA) (Items 3, 4, 8, and 11), and Friends (FR) (Items 6, 7, 9, and 12). In the revised version, Cronbach’s alpha was.92, and for the sub-scales, it was.91,.88, and.92 for FR, FA, and SO respectively (28). Those who score higher, mean that they have greater social support perceived by an individual; the total possible score ranges from 12 to 84.

#### Parents

This study refers to all individuals who hold significant influence in an adolescent’s life and offer unpaid care, irrespective of being biologically related. This encompasses not only biological parents (both mother and father) but also grandparents, older relatives, and other caregivers who provide support without financial compensation (30).

Knowledge of sexual and reproductive health (SRH) rights was evaluated using 24 binary items, with responses coded as 1 for “yes” (correct) and 0 for “no” (incorrect). Each participant’s total score was calculated by summing these 24 items, and the mean score was computed to represent the proportion of correct answers. To categorize knowledge levels, participants scoring at or above the mean were classified as having “good knowledge,” while those scoring below the mean were classified as having “poor knowledge,” consistent with methodologies used in similar studies (31, 32).

#### Paternal monitoring

It was assessed using five items adapted from the study conducted in Harar, Ethiopia (33). These items measured how often the parents know what he/she does in his/her free time; where he/she goes at night; who his/her friends are; how he/she spends his/her money; and where he/she goes after school. The scale had a good internal consistency of Cronbach a greater than 0.71 (33). Responses ranged from ‘not at all’ (coded as 1) to ‘always’ (coded as 4). Responses will sum to form a continuously treated scale with a range of 5–20. Then, the 33rd and 67th percentiles of the composite scores of paternal monitoring were used to categorize them into low (<33rd percentile), medium (between 33rd and 67th percentiles; inclusive), and high (67th percentile) (33).

### Data collection and management

Data were collected by twelve trained health professionals with experience in data collection using KoBoToolbox software were selected as data collectors. They were closely supervised by three supervisors who had greater experience in data collection. When selecting data collectors and supervisors, their ability to communicate in the local language and data collection experiences were used as a criterion. The principal investigator provides a two-day extensive training for supervisors and data collectors. The focus of the training is on administering the questionnaire, maintaining confidentiality and privacy, and neutrality during interviews on sensitive topics. After the two-day training, a pretest was conducted with 60 adolescents (5%) in Chencha District, Gamo Zone, who were not selected for final data collection. After the pilot study, content, and face validation, necessary changes were made, such as removing confusing and unnecessary questions. The final version of the questionnaire was then uploaded to the KoBoToolbox software. A structured questionnaire was used to collect the data. This tool (questionnaire) was created after reviewing previous studies (24–26, 28, 29, 31–34). The questionnaire was originally developed in English and translated into the local language (Amharic). The data collection tool was face and content validated by reproductive health experts before actual data collection. Each time we visited a village, we revisited the house to get previously absent adolescents to maximize participation rates. The principal investigator and supervisors oversaw the entire data collection process and checked the data for completeness daily. Before the data collectors send the collected data to the center supervisors check the completeness of the questionnaires. In addition, the principal investigator regularly reviewed the files sent to the center by each data collector.

### Adaptation and validation of the MSPSS

To measure perceived social support among Ethiopian adolescents, we adapted the revised Multidimensional Scale of Perceived Social Support (MSPSS). The adaptation process involved reviewing the original scale thoroughly, consulting with local experts and stakeholders, translating the scale forward and backward, and refining it through cognitive interviews with adolescents. A pretest was conducted to evaluate the scale’s functionality and make necessary adjustments.

For validating and ensuring the reliability of the adapted scale, several methods were employed. We assessed internal consistency using Cronbach’s alpha, which yielded a high value of 0.8429, indicating strong coherence among the 12 items. The average inter-item covariance was 0.8467, supporting the scale’s consistency. Test-retest reliability was confirmed by administering the scale to a subset of participants on two separate occasions, demonstrating stability over time.

Content validity was established through a systematic process. Measurement instruments were developed based on an extensive literature review and theoretical framework. Feedback from a panel of experts—including researchers, practitioners, and clinicians—was used to review the relevance and clarity of the items. This feedback led to refinements in item wording and alignment with the study’s objectives.

Exploratory and confirmatory factor analyses (CFA) were conducted to assess construct validity. The CFA evaluated a model with three latent factors and showed an adequate fit. Despite a significant chi-square test (χ²(54) = 120.43, p < 0.001), which is typical in large samples, fit indices indicated a good model fit: Comparative Fit Index (CFI) of 0.95, Tucker-Lewis Index (TLI) of 0.94, Root Mean Square Error of Approximation (RMSEA) of 0.07 (90% CI: 0.05 to 0.09), and Standardized Root Mean Square Residual (SRMR) of 0.05. Standardized factor loadings ranged from 0.60 to 0.85 and were statistically significant (p < 0.01), confirming that the observed variables were strong indicators of their latent factors. Minor modifications, such as correlating error terms between items 5 and 6, slightly improved the fit indices. Overall, the adapted MSPSS demonstrated good fit and alignment with theoretical expectations.

### Data analysis and management

The data collected for this study were rigorously cleaned, processed, and analyzed using STATA version 14.0. We first computed descriptive statistics, including frequencies, percentages, means, and standard deviations, which were presented through comprehensive text narratives, graphs, and tables. To assess the reliability of our measurements, we calculated Cronbach’s alpha for each composite variable, with values exceeding 0.70 indicating strong internal consistency.

To explore the relationships between independent variables and the dependent variable, perceived social support, we began with univariate analyses to examine individual associations. Variable selection for the final model was guided by theoretical frameworks and prior research. Multiple linear regression analysis was then employed to control for confounding factors.

The final model was refined through stepwise regression, integrating both statistical criteria (e.g., p-values) and theoretical relevance. We used β coefficients to quantify the strength and direction of associations: positive β coefficients denoted a positive relationship with perceived social support, while negative β coefficients indicated a negative relationship. Precision of these estimates was evaluated using 95% confidence intervals (CIs), providing a range within which the true population parameter is likely to fall. Statistical significance was established at p < 0.05, with p-values below this threshold considered significant.

To evaluate model fit, we utilized R-squared and adjusted R-squared metrics to determine the proportion of variance in the dependent variable explained by the independent variables, with adjusted R-squared offering a more accurate measure in the context of multiple predictors. Additionally, we assessed residual plots to confirm that key model assumptions—linearity (the relationship between independent and dependent variables), normality of residuals (ensuring they are approximately normally distributed), and homoscedasticity (constant variance of residuals across levels of independent variables) were assessed.

## Results

### Socio-demographic characteristics and parenting practices of adolescents

The study achieved a 99% response rate. It reveals that a slight majority of adolescents are female, comprising 56.31% of the sample, while males account for 43.69%. The mean age of respondents is 15.01 years (± 2.69 SD). Adolescents aged 15 to 19 years make up 57.59% of the sample (675 individuals), compared to 42.41% (497 individuals) aged 10 to 14 years.

In terms of schooling status, the majority are enrolled in school, with 1,022 adolescents (87.20%), while 150 individuals (12.80%) are out of school. Regarding residential distribution, 58.79% of adolescents reside in urban areas.

Assessment of knowledge about sexual and reproductive health (SRH) rights shows a relatively balanced distribution: 590 adolescents (50.34%) have poor knowledge and 582 (49.66%) have good knowledge. Employment status indicates that 293 adolescents (24.9%) have engaged in paid work within the last 12 months, while the majority, 882 individuals (75.1%), has not.

Perceived risks for SRH problems are notable, with 308 adolescents (26.3%) perceiving such risks, while 864 (73.7%) do not. Examination of living arrangements reveals that most adolescents live with both biological parents (871 or 74.32%), while others reside with a single biological parent or other caregivers. Parental monitoring varies, with 419 adolescents (35.75%) reporting low levels, 378 (32.25%) reporting medium levels, and 375 (32.00%) reporting high levels. Lastly, perceived economic status of households indicates that 221 adolescents (18.9%) are from poor households, 850 (72.5%) from medium households, and 101 (8.6%) from wealthy households (Refer to **Table 1**).

**Table 1:** Socio-demographic characteristics and parenting practices of adolescents in Gamo Zone, Southern Ethiopia, 2024.

Characteristics of the respondents	Categories	N (%)
Sex of the adolescent	Female	660(56.31)
	Male	512(43.69)
Adolescent age in years	10 to 14 years	497 (42.41)
	15 to 19 years	675 (57.59)
Marital Status	Married	35(2.99)
	Single	1137(97.01)
Schooling of the adolescents	In-school	1022(87.20)
	Out-of-school	150(12.80)
Place of residence	Urban	689(58.79)
	Rural	483(41.21)
Adolescent Knowledge about SRH rights	Poor Knowledge	590(50.34)
	Good Knowledge	582(49.66)
Paid work in the last 12 months	Yes	293(24.9)
	No	882(75.1)
Perceived risks for SRH problems	Yes	308(26.3)
	No	864(73.7)
Adolescent living arrangements	With biological mother and father	871(74.32)
	With the biological mother alone	104(8.87)
	With biological father alone	24(2.05)
	With a sister or brother	43(3.67)
	With guardian/caregivers	99(8.45)
	With others(+)	31(2.65)
Parental monitoring	Low	419(35.75%)
	Medium	378(32.25%)
	High	375(32.00%)
The perceived economic status of household	Poor	221(18.9)
	Medium	850(72.5)
	Wealthy	101(8.6)

Living with others (+) includes individuals residing with members beyond the nuclear family, such as extended family members (e.g., grandparents, aunts, uncles, or cousins), non-relatives (e.g., family friends or guardians), and those in foster or group homes, whether temporarily or permanently.

### Perceived social support

The results regarding perceived social support among the respondents reveal a diverse distribution across different levels. Among the surveyed individuals, 411(35.07%) reported experiencing low levels of social support. Meanwhile, 371(31.66%) adolescents reported receiving medium levels of social support. In contrast, a slightly higher proportion, 390(33.28%) adolescents reported experiencing high levels of social support. **Table 2** displays the descriptive statistics for perceived social support across different sub-dimensions of the scale. The mean scores along with their standard deviations (SD) are presented for each dimension, namely family, friends, and significant others. For the family dimension, the mean score was 22.44 with a standard deviation of 4.54, ranging from 5 to 28. In the friends’ dimension, the mean score was slightly lower at 17.75, with a higher standard deviation of 5.94, and scores varying between 4 and 28. The significant others dimension had a mean score of 16.73, with the highest standard deviation of 6.72, also spanning from 4 to 28. The total support score, which combines the scores from all dimensions, had a mean of 57.02 and a standard deviation of 12.68, with scores ranging from 19 to 84. (Refer to **Table 2**).

**Table 2:** Descriptive statistics of perceived social support among adolescents in the Gamo Zone, Southern Ethiopia, 2024.

Sub Dimensions of Scale	Mean ± SD	Min - Max
Family	22.44 ± 4.54	5 to 28
Friends	17.75 ± 5.94	4 to 28
Significant others	16.73± 6.72	4 to 28
Total support	57.02 ± 12.68	19 to 84

### Factors associated with perceived social

#### Model fit indices

The model demonstrated a strong fit, as reflected by an Adjusted R² value of 0.372. This suggests that 37.2% of the variance in the outcome variable can be explained by the predictor variables included in the model. The overall F-statistic was significant (F(10, 1161) = 10.10, p < 0.001), indicating that the regression model significantly improves upon a model with no predictors. This significant F-statistic reinforces the model’s effectiveness in capturing the relationship between the predictors and the outcome variable.

Furthermore, the R² value of 0.38 indicates that 38% of the variance in the outcome variable is explained by the model. This high R² value demonstrates the model’s substantial explanatory power, reflecting its ability to account for a considerable portion of the variability in the outcome. Together, these fit indices suggest that the model is robust and provides a meaningful representation of the relationships among the variables in the analysis.

### Model assumptions

#### Model assumptions

In our analysis, we rigorously addressed the core assumptions of linear regression: normality of residuals, absence of multicollinearity, and linear relationships between variables. We conducted thorough preliminary data checks to evaluate the distribution characteristics of the variables.

Given that our social support data were ordinal and did not strictly meet the normality assumption, we implemented a normalization strategy to address this limitation. Social support was measured using 12 Likert scale items, each ranging from 1 (very strongly disagree) to 7 (very strongly agree). Recognizing that Likert scale data are inherently ordinal and often fail to meet normality requirements, we applied a normalization transformation to the composite score of these Likert items. This transformation aimed to approximate interval-level measurement, thereby justifying the use of parametric methods like linear regression.

Our approach is supported by previous research, such as studies by Norman (2010) and Carifio & Perla (2008), which argue that combining multiple Likert items into a composite score can reasonably approximate interval-level measurement, making parametric methods suitable. The transformation not only improved the model’s fit but also ensured adherence to critical regression assumptions, enhancing the validity and reliability of our analysis.

The normalization transformation led to significant improvements in model performance. The R-squared value, which represents the proportion of variance explained by the model, increased from 0.62 in the untransformed model to 0.75 in the transformed model, indicating a stronger relationship between the variables. Additionally, the Akaike Information Criterion (AIC), which penalizes model complexity, decreased from 128 to 110, reflecting a more efficient model fit with reduced residual errors. These results underscore the benefits of transforming the Likert scale data, enhancing both the accuracy and interpretability of the model.

#### Assessment of assumptions

Following the transformation, we reassessed the model to ensure that the assumptions required for linear regression were met. To evaluate the normality of the residuals, we employed three graphical methods: kernel density estimation (kdensity), quantile-quantile plot (Q-Q plot), and cumulative probability plot (P-P plot).

**Figure 2:**
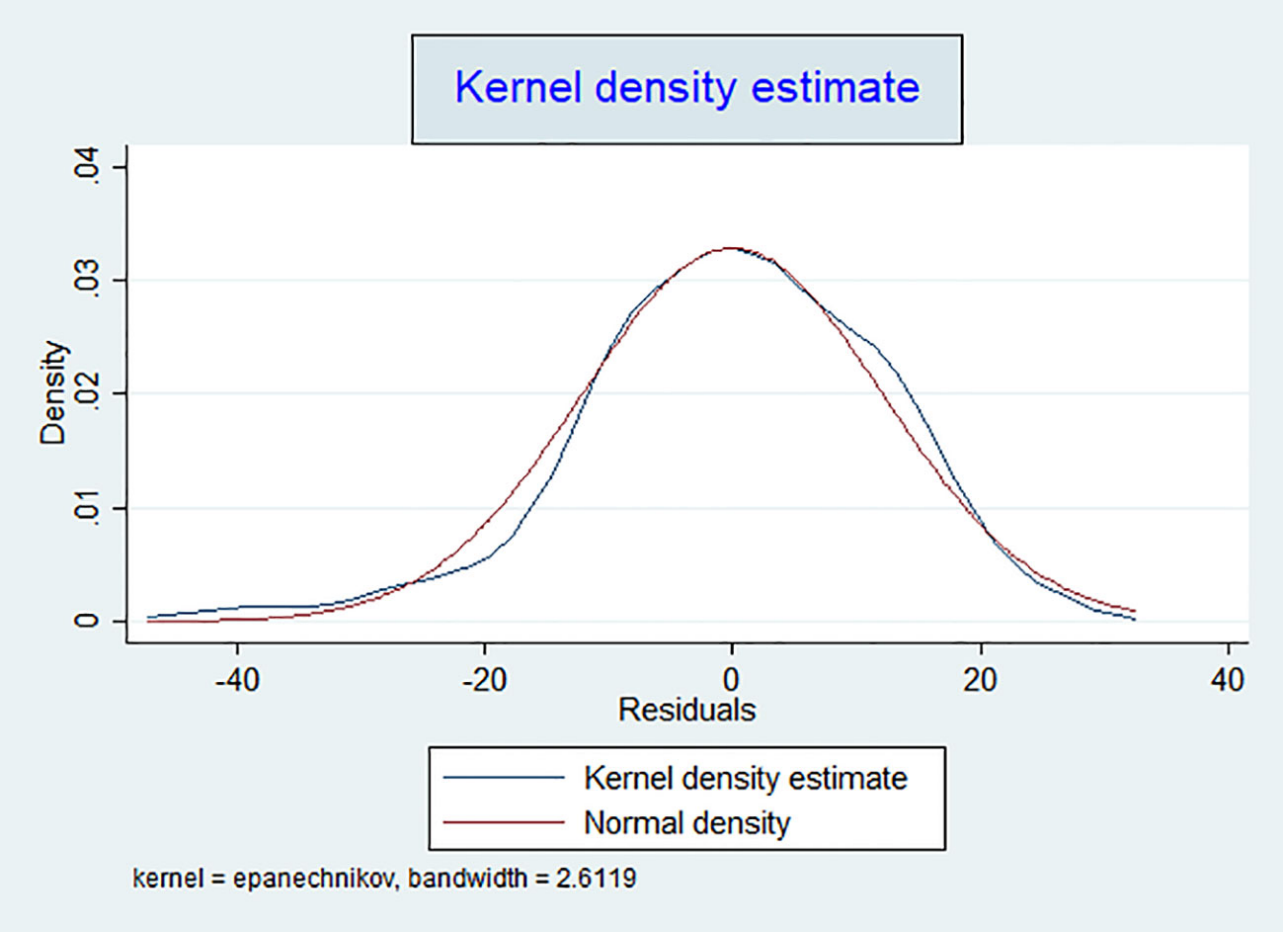
Kernel density plot of transformed residuals for assessing distribution in the linear regression model.

**Figure 3:**
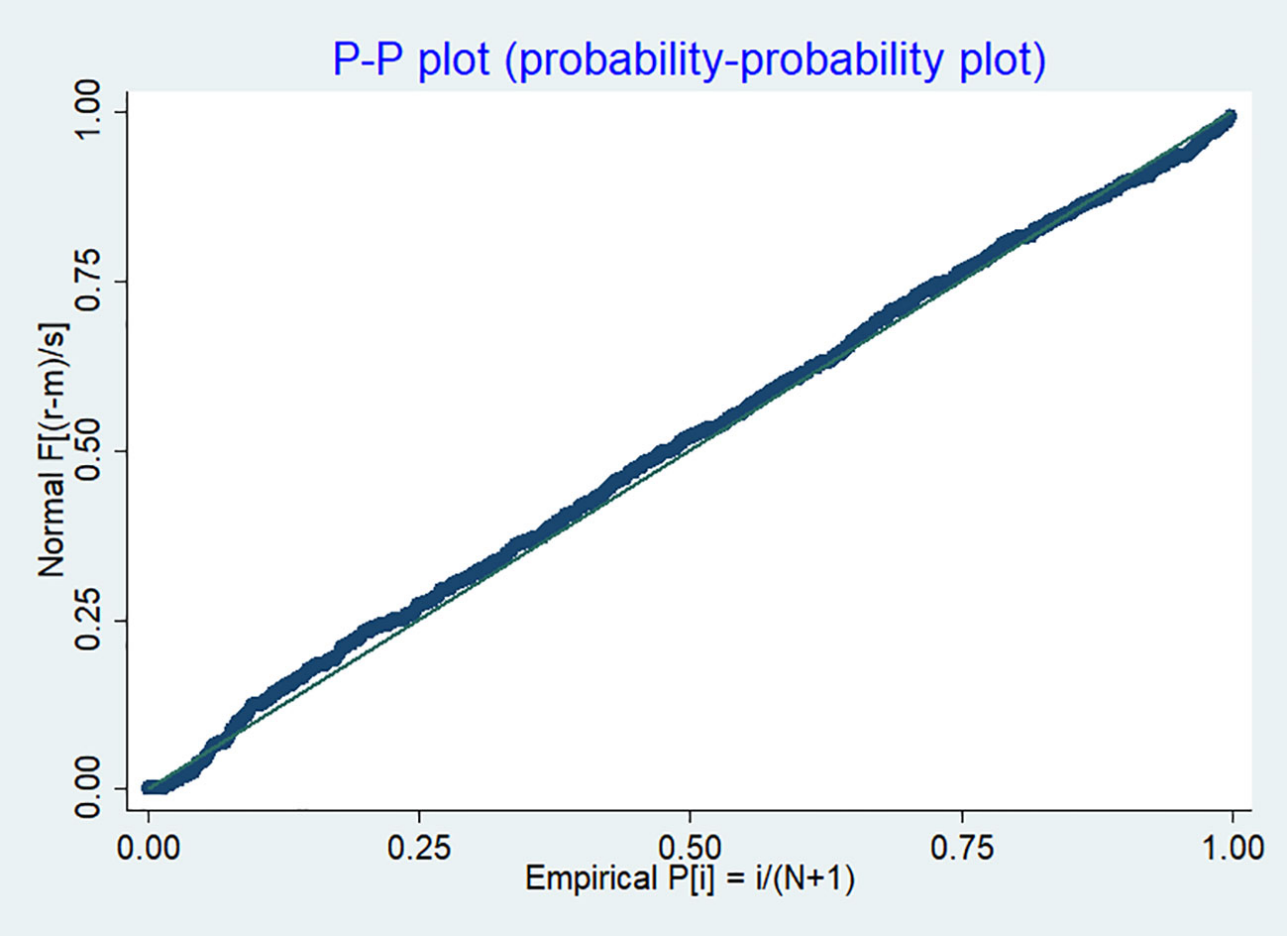
P-P plot of transformed residuals for assessing normality in the linear regression model.

The kernel density plot compared the distribution of residuals to a normal distribution curve (**Figure 2**). A well-aligned curve suggests that the residuals follow a normal distribution. The P-P plot assessed the cumulative distribution of the residuals against the expected normal distribution, with residuals aligning with the diagonal line indicating normality (**Figure 3**).

**Figure 4:**
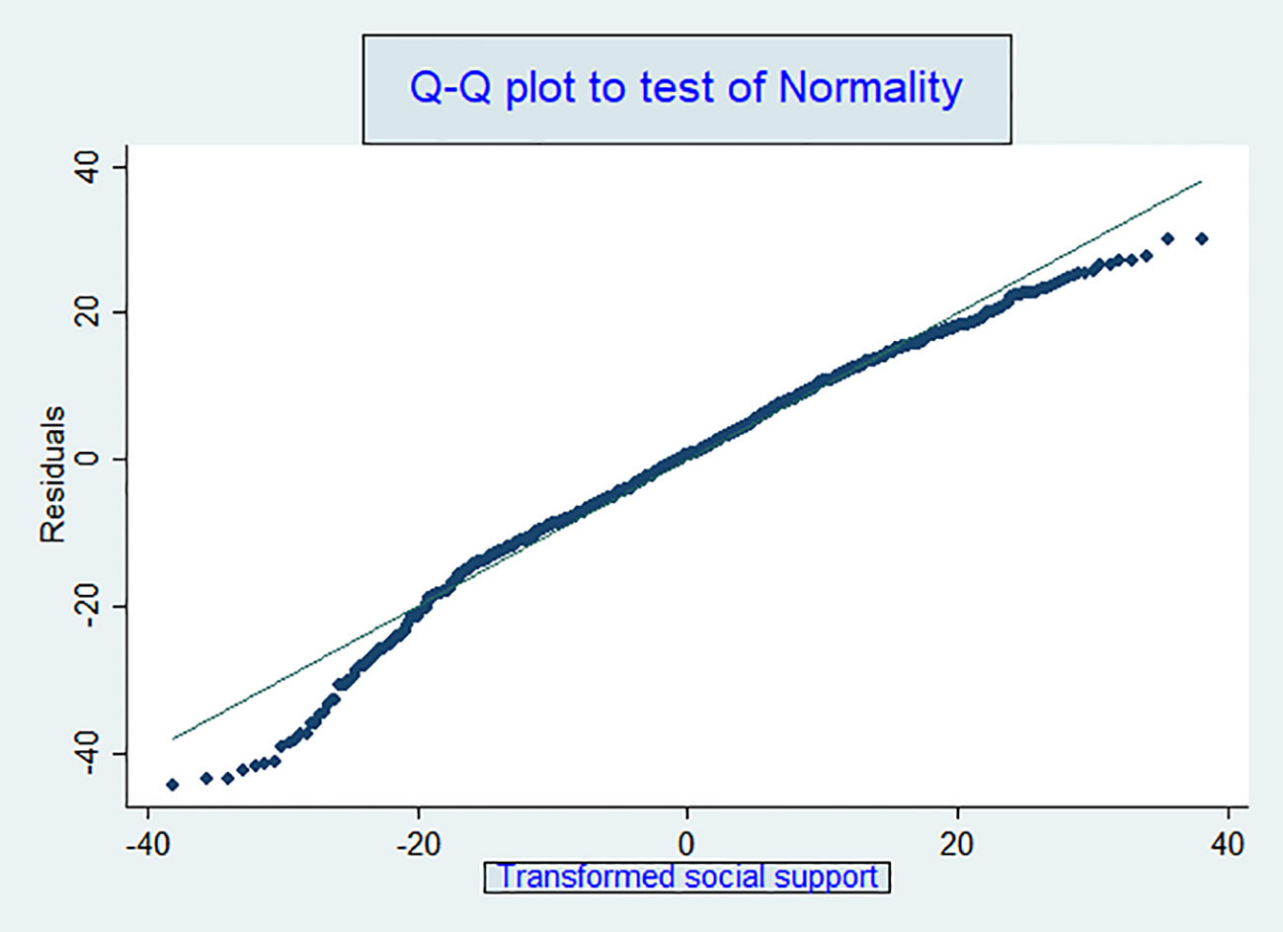
Q-Q plot of residuals for assessing normality in the linear regression model.

The Q-Q plot further validated the effectiveness of the transformation in improving data normality. By comparing the quantiles of the observed data to those of a theoretical normal distribution, the Q-Q plot (**Figure 4**) showed that most data points closely followed the 45-degree reference line, indicating that the transformed residuals approximate a normal distribution. Minor deviations at the tails were minimal and did not significantly impact the overall normality of the data. The Shapiro-Wilk test, with a p-value greater than 0.05, corroborated this finding, confirming that the transformed data adequately approximate a normal distribution. This reinforces the appropriateness of using parametric methods such as linear regression in our analysis.

To assess the homoscedasticity assumption (constant variance of residuals), we performed a Breusch-Pagan/Cook-Weisberg test. The test yielded a chi-square value of 0.13 with 1 degree of freedom and a p-value of 0.7160. Since the p-value exceeds the conventional significance level of 0.05, we do not reject the null hypothesis, indicating no evidence of heteroscedasticity. This was further supported by the residual plot, which showed consistent variance across residuals. These findings suggest that the data transformation effectively addressed issues related to skewness and variance, resulting in a more robust model. Additionally, the transformation improved adherence to the linearity assumption, ensuring accurate representation of the relationships between independent and dependent variables.

We also evaluated collinearity among predictor variables using variance inflation factor (VIF) tests. The VIF results indicated an overall mean of 1.31, with a minimum of 1.03 and a maximum of 1.58—well below the commonly accepted threshold of 10. These results confirm that multicollinearity is not a significant concern in our model. By meeting these key assumptions, we have ensured the validity, reliability, and robustness of our regression analysis, thereby strengthening the credibility of our findings.

The linear regression analysis finding shows that among the various factors assessed, adolescents living with their biological parents reported a perceived social support level that was 4.17 units higher (β = 4.17, 95% CI: 2.00 to 6.34) compared to those not living with their biological parents. This suggests a strong relationship between living with biological parents and higher levels of perceived social support. Adolescents who had engaged in paid work in the past 12 months showed a perceived social support level 3.43 units higher (β = 3.43, 95% CI: 1.39 to 5.46) than those who had not worked for pay during the same period, indicating that paid work is also associated with increased perceived social support. Those with high levels of parental monitoring reported a perceived social support level 3.03 units higher (β = 3.03, 95% CI: 1.23 to 4.82) compared to those with low levels of parental monitoring, reflecting the positive impact of parental involvement on perceived social support. Adolescents who perceived risks for SRH problems had a perceived social support level 2.76 units higher (β = 2.76, 95% CI: 0.84 to 4.69) compared to those who did not perceive such risks, suggesting that awareness of risks may also enhance perceived social support. Additionally, each one-unit increase in adolescent knowledge about sexual and reproductive health (SRH) rights was associated with a 2.46-unit increase in perceived social support (β = 2.46, 95% CI: 0.89 to 4.02), highlighting a positive correlation between SRH rights knowledge and perceived social support. Lastly, adolescents residing in rural areas reported a perceived social support level 1.56 units higher (β = 1.56, 95% CI: 0.16 to 3.11) compared to those living in urban areas, while controlling for other variables (see **Table 3**).

**Table 3:** Factors associated with perceived social support among adolescents in the Gamo Zone, Southern Ethiopia, 2024.

Variables	Categories	Crude β (95% CI)	Adjusted β (95% CI)	P value
Sex of the adolescent	Female	0.31(-1.16,1.78)	0.32(-0.12,1.75)	0.665
	Male	**Ref**	**Ref**	
Adolescent age in years	10 to 14 years	**Ref**	**Ref**	
	15 to 19 years	3.45(1.99,4.91)	1.42(-0.22,3.07)	0.090
Schooling of the adolescents	In-school	**Ref**	**Ref**	
	Out-of-school	0.38(-1.79,2.56)	1.14(-1.16,3.44)	0.331
Place of residence	Urban	**Ref**	**Ref**	
	Rural	0.04(-1.43,1.52)	***1.56(0.16,3.11)***	0.048**
Adolescent Knowledge about SRH rights	Poor Knowledge		**Ref**	
	Good Knowledge	3.98(2.55,5.42)	***2.46(0.89,4.02)***	0.002***
Paid work in the last 12 months	Yes	5.87(4.22,7.51)	***3.43(1.39,5.46)***	0.001**
	No	**Ref**	**Ref**	
Perceived risks for SRH problems	Yes	4.92(3.29,6.54)	***2.76(0.84,4.69)***	0.005**
	No	**Ref**	**Ref**	
Adolescent living arrangements	Lived with their biological parents	3.55(1.51,5.59)	***4.17(2.00,6.34)***	0.0001**
	Not living with their parents	**Ref**	**Ref**	
Parental monitoring	Low	**Ref**	**Ref**	
	Medium	-0.46(-2.23,1.30)	0.23(-1.52,1.99)	0.793
	High	1.16(-0.61,2.93)	**3.03(1.23,4.82)**	0.001**

** *p* <.05; Ref, reference category. The bold values indicate statistically significant variables.

## Discussion

This study aimed to identify factors associated with adolescents’ perceived social support. Understanding the factors that contribute to adolescents’ perceptions of social support is essential for developing targeted interventions to promote their well-being and resilience. The findings reveal significant positive associations between living arrangements, employment status, parental monitoring, perceived risks for SRH problems, and knowledge about SRH rights, geographical context, and adolescent perceived social support. These findings highlight the importance of considering a comprehensive range of factors when designing interventions to promote adolescent well-being.

The findings of this study reveal that there is a significant positive association between adolescents’ living situations and their perceived social support. This finding aligns with findings from previous studies conducted in diverse contexts, indicating a significant positive association between adolescents’ living situations and their perceived social support (35, 36). This suggests that adolescents who live in certain family structures may experience higher levels of social support compared to others. This might be because adolescents who live with their biological parents often have daily interactions with family members, providing opportunities for emotional connection, sharing of experiences, and receiving guidance and support (37). This supportive environment can contribute to adolescents feeling more valued, understood, and connected (38), which in turn may enhance their perception of social support. However, it is essential to acknowledge potential confounding factors that may influence this relationship, such as the quality of parent-child relationships, household dynamics, and socio-economic status. Future research could explore these factors in more depth to better understand the mechanisms underlying the association between living arrangements and perceived social support among adolescents. This finding highlights the significance of investing in nurturing and maintaining positive familial relationships is crucial for promoting adolescents’ perception of social support and fostering their healthy development. In addition, these results underscore the vital role of community-based interventions in offering resources and support to families, which can help strengthen social support networks for adolescents in various living arrangements. Community-based programs are effective in enhancing family functioning and providing adolescents with a supportive environment that fosters resilience and well-being (39, 40).

This study’s results revealed a significant positive relationship between adolescents’ involvementin paid employment over the past year and their perceived social support. This finding aligns with previous research examining the relationship between adolescent employment and various perceived social supports, which found a significant positive association between adolescents’ engagement in paid employment and their perceived social support (41). Similarly, another study explored the impact of adolescent employment on diverse dimensions of psychological well-being, highlighting a notable positive relationship between adolescents’ participation in paid work and their perceived social support (42). Furthermore, findings from a longitudinal study, identified a consistent positive correlation between adolescents’ involvement in paid employment and their perceived social support, indicating a sustained effect over time (43). One possible explanation for this discovery is that employment provides adolescents with opportunities for social interaction and networking, fostering supportive relationships with peers and colleagues (44). The sense of achievement and autonomy from employment may enhance adolescents’ overall social well-being, while financial independence can reduce family financial strain and strengthen family support systems (45). However, adolescents’ experiences with employment can vary based on factors like work type and environment (46). In Ethiopia and other African contexts, employment can impact social support uniquely, influencing family dynamics and adolescent well-being (47, 48). Future research should explore these nuances to provide a comprehensive understanding of the relationship between adolescent employment and perceived social support.

This study finding revealed that there significant positive association between higher levels of parental monitoring and increased perceived social support among adolescents. This finding is consistent with previous study findings (6, 49, 50). This positive relationship highlights the significant role that parental involvement and supervision play in adolescents’ social development. One possible explanation for this finding is that parental monitoring fosters a supportive and communicative parent-child relationship, wherein adolescents feel comfortable seeking guidance and support from their parents. This open communication channel may lead to greater emotional support and a sense of security among adolescents, contributing to their overall perception of social support. Furthermore, parental monitoring may also facilitate the establishment of positive social connections outside the family unit. Adolescents who feel supported and guided by their parents may be more likely to engage in healthy social activities and form meaningful relationships with peers. These positive social interactions can further enhance adolescents’ perceived social support by providing them with additional sources of emotional and instrumental support (50). Moreover, parental monitoring can serve as a protective factor against various risk behaviors and negative social influences (6, 51). However, it’s essential to recognize that the relationship between parental monitoring and perceived social support may be influenced by various contextual factors, such as family dynamics, cultural norms, and individual characteristics. Additionally, excessive parental control or intrusive monitoring may have detrimental effects on adolescents’ autonomy and independence, potentially undermining their perceived social support. Future research should further deeper into understanding the mechanisms underlying the relationship between parental monitoring and perceived social support to inform effective interventions and support strategies for adolescents and their families. Furthermore, longitudinal studies are warranted to examine the long-term effects of parental monitoring on adolescents’ social support trajectories, providing valuable insights into its implications for adolescent well-being throughout different stages of development.

A significant positive association was found between adolescents’ perceptions of SRH risks, their knowledge about SRH rights, and their perceived social support. Previous research findings also show that there is a significant positive association between adolescents’ perceptions of SRH risks and their perceived social support over time (52). Furthermore, another investigation demonstrates that elevated levels of perceived social support correspond to decreased involvement in risky sexual behaviors (53). Similarly, qualitative research findings suggest that adolescents with heightened levels of social support often perceive fewer SRH risks and demonstrate healthier decision-making behaviors (54). Additionally, a systematic review synthesizing various findings also underscores a consistent positive relationship between perceived social support and diminished engagement in risky sexual behaviors among adolescents (55). This might be because adolescents who perceive stronger social support networks may be more inclined to seek out information and assistance regarding SRH concerns, fostering heightened awareness of potential risks and a proactive stance in addressing them (56). Moreover, the observed link between adolescents’ awareness of SRH rights and their perceived social support implies that access to information concerning one’s SRH rights can fortify adolescents’ sense of empowerment and autonomy (57). Equipped with this knowledge, adolescents are better positioned to make informed decisions about their sexual and reproductive health (SRH), access appropriate support systems, and advocate for their rights within their social circles and broader communities (58, 59). These findings underscore the importance of acknowledging and addressing SRH concerns within adolescent social networks. Creating supportive environments where adolescents feel at ease discussing SRH topics openly and seeking support when necessary is vital for promoting informed decision-making and positive SRH outcomes (59). Additional longitudinal research is required to explore the causal connections between perceived SRH risks and social support across time (60).

Moreover, the results indicated a notable discrepancy in perceived social support among adolescents residing in rural and urban areas. This difference in perceived social support may be attributed to the tightly-knit nature of rural communities, which fosters stronger social bonds and support networks among adolescents (61, 62). In rural settings, individuals often have closer proximity to their neighbors and community members, facilitating frequent interactions and the cultivation of supportive relationships (5, 61). Additionally, rural environments offer distinctive opportunities for adolescents to engage in communal activities and shared experiences, which further enhance their sense of social support. Such activities may encompass participation in community events, religious gatherings, or local customs that foster a sense of belonging and interconnectedness (5). These findings underscore the significance of considering the distinct social contexts of various settings when formulating interventions aimed at promoting adolescent health and well-being.

### Limitations of the study

This study has several limitations. Firstly, it is cross-sectional in nature, which restricts the ability to establish causal relationships between variables. Additionally, potential confounding factors, such as the quality of parent-child relationships, household dynamics, and socio-economic status, were not fully accounted for, which may influence the study’s findings and affect the interpretation of the results. Another limitation is the exclusion of participants with hearing problems, which may limit the generalizability of the findings to the broader adolescent population.

## Conclusion

In conclusion, this study underscores the significant influence of various factors on adolescents’ perceived social support. Factors such as living arrangements, employment status, parental monitoring, perceived risks for SRH problems, knowledge about SRH rights, and geographic location were identified as key predictors of perceived social support. Notably, adolescents living with their biological parents reported notably higher levels of perceived social support. Similarly, involvement in paid employment over the past year was associated with increased perceived social support, suggesting potential benefits of financial independence and workplace interactions. Additionally, stronger parental monitoring was linked to greater perceived social support among adolescents. Moreover, adolescents who perceived risks for SRH problems, as well as those with a good understanding of SRH rights, reported heightened levels of perceived social support, indicating the influence of health awareness and perceived vulnerability on social support perceptions. Furthermore, geographic location played a role, with adolescents in rural areas reporting higher levels of perceived social support compared to their urban counterparts, emphasizing the importance of considering environmental factors in understanding adolescents’ social support dynamics.

## Recommendations

Drawing from the findings of this study, the following recommendations are proposed. By acting upon these recommendations, stakeholders can play an essential role in fostering adolescent well-being and resilience by strengthening social support networks.

### Promote family-based support systems

The significant association between living with biological parents and higher perceived social support suggests the importance of maintaining strong family connections. Programs and policies that support family unity and address challenges faced by adolescents not living with their biological parents could be beneficial. Community and government initiatives should focus on strengthening family support structures to improve adolescents’ social support networks.

### Encourage paid work opportunities

The positive relationship between paid work and perceived social support highlights the potential benefits of employment for adolescents. Efforts should be made to create and expand job opportunities for young people, especially those who might benefit from additional financial independence and social interactions associated with work. Such initiatives could enhance their overall social support and development.

### Support parental involvement

The findings underscore the value of high levels of parental monitoring in increasing perceived social support. Programs aimed at educating and empowering parents to be more actively involved in their adolescents’ lives could strengthen the support system available to young people. Schools and community organizations should consider offering workshops and resources to help parents engage more effectively.

### Enhance SRH awareness and education

The finding that adolescents who perceived risks for SRH problems and those with higher knowledge of SRH rights reported greater perceived social support highlights the importance of both increasing awareness of SRH risks and enhancing SRH rights education. To leverage these insights, public health campaigns and school-based programs should focus on comprehensive SRH education that includes raising awareness about potential risks and informing adolescents about their SRH rights. By integrating these educational components, we can empower adolescents with essential knowledge and resources, thereby improving their social support networks and overall well-being.

### Address geographic disparities

The finding from our study that adolescents in rural areas reported higher levels of perceived social support compared to their urban counterparts suggests that rural communities may have unique support structures contributing to this positive outcome. To address this disparity, further research should focus on identifying the specific factors and mechanisms in rural areas that enhance social support. Urban areas could benefit from these insights by adapting and implementing successful support strategies observed in rural settings. By understanding and replicating these effective practices, urban communities can work towards improving social support levels for adolescents and bridging the gap between different geographic settings.

## Data Availability

All the data generated or analyzed during the study was included in this manuscript. However, the de-identified datasets used in the reported study are available upon reasonable request from the corresponding author.
